# A qualitative exploration of the healthcare challenges and pharmaceutical care needs of people with Parkinson’s and their caregivers

**DOI:** 10.1007/s11096-021-01312-4

**Published:** 2021-07-27

**Authors:** Sabrina Anne Jacob, Zhi Jean Wong, Wing Loong Cheong, Elizabeth Yie-Chuen Chong, Yin Xuan Wong, Sara Lai Heong Lew

**Affiliations:** 1 School of Pharmacy, Monash University Malaysia, Jalan Lagoon Selatan, 47500 Bandar Sunway, Selangor Malaysia; 2 Strathclyde Institute of Pharmacy and Biomedical Sciences, University of Strathclyde, 161 Cathedral St, Glasgow, G4 0RE Scotland; 3 Jeffrey Cheah School of Medicine and Health Sciences, Monash University Malaysia, Jalan Lagoon Selatan, 47500 Bandar Sunway, Selangor Malaysia; 4 Malaysian Parkinson’s Disease Association, No. 35, Jalan Nyaman 10, Happy Garden, Jalan Kelang Lama, 58200 Kuala Lumpur, Malaysia

**Keywords:** Medication Adherence, Parkinson’s Disease, People Centred Care, Pharmaceutical Care, Qualitative Research

## Abstract

*Background* People with Parkinson’s are at higher risk of healthcare and pharmaceutical care issues. *Objective* To determine the healthcare challenges, pharmaceutical care needs, and perceived need of a pharmacist-run clinic by people with Parkinson’s and their caregivers. *Setting* Malaysian Parkinson’s Disease Association. *Method* A focus group discussion adopting a descriptive qualitative approach was conducted involving people with Parkinson’s and their caregivers. A semi-structured interview guide was used to determine the challenges they faced with their medications and healthcare system, their pharmaceutical care needs, and their views on a pharmacist-run clinic. Data was thematically analysed. *Main outcome measure*: Healthcare challenges faced by people with Parkinson’s and caregivers along with their pharmaceutical care needs and perceived need of a pharmacist-run clinic. *Results* Nine people with Parkinson’s and four caregivers participated. Six themes were developed: (1) “It’s very personalised”: the need for self-experimentation, (2) “Managing it is quite difficult”: challenges with medication, (3) “The doctor has no time for you”: challenges with healthcare providers, (4) “Nobody can do it except me”: challenges faced by caregivers, (5) “It becomes a burden”: impact on quality of life, and (6) “Lack of consistency could be counterproductive”: views on pharmacist-run clinic. *Conclusion* The provision of pharmaceutical care services by pharmacists could help overcome issues people with Parkinson’s face, however there is a need for them to first see pharmacists in their expanded roles and change their limited perception of pharmacists. This can be achieved through integration of pharmacists within multidisciplinary teams in specialist clinics which they frequent.

## Impacts on Practice

- Healthcare providers should make an effort to identify people with Parkinson’s who practice unsupervised self-experimentation, explore their reasons, and work with people with Parkinson’s and their carers to prevent future such practices.
- Better communication and people-centred care should be adopted by healthcare providers to prevent and alleviate the challenges people with Parkinson’s and carers face with medications and the healthcare system.
- Pharmacists should assume a greater role within multidisciplinary teams in specialist clinics so people with Parkinson’s are aware of their expanded roles.

## Introduction

Parkinson’s disease (PD) is the most common movement disorder, reported in approximately 6.2 million people worldwide [1]. People with Parkinson’s (PwP) are at risk of experiencing drug-related problems (DRPs) due to their advanced age, comorbidities, polypharmacy, and the multifaceted and complex nature of the disorder [2, 3]. In a study involving PwP, 160 study participants experienced 238 DRPs over a 1-year period [3]. Only 39% of PwP are adherent to their medication [4], and this nonadherence contributes to the development of complicated non-motor symptoms [5, 6], and poorer quality of life as the disease progresses [7]. This, in turn, increases the burden and cost of care owing to the need for more complicated dosing regimens, increased doses, and the need for new medications to mitigate the worsening of symptoms [4, 8, 9].

Pharmaceutical care (PC) has been shown to significantly improve patient health and the quality of treatment outcomes [4]. At the core of PC is people-centred care, and indeed there have been calls for more people-centred care in PD [10, 11]. In one study, PD-specific medication use reviews undertaken by pharmacists resulted in improved adherence, and identification of DRPs. More than 80% of participants also felt their knowledge on their medication had improved [12]. Elsewhere a medication therapy management service by pharmacists resulted in a positive improvement in patients’ non-motor symptoms and a significant decrease in DRPs [4].

### Aim

The Malaysian Parkinson’s Disease Association (MPDA) is a non-governmental organization which provides support services to more than 1000 PwP, and their caregivers, who are members of the Association. With the intent of establishing a pharmacist-run clinic within the MPDA, a study was undertaken to explore the PC needs and challenges currently faced by PwP and their caregivers.

### Ethics approval

The research protocol was approved by the Monash University Human Research Ethics Committee (MUHREC; project number 0738).

## Method

### Design

A focus group discussion (FGD) adopting a descriptive qualitative approach was conducted. A community-based participatory research approach was adopted [13], where the president of the MPDA (SL) was involved in the development of the FGD guide with the researchers, and recruitment of participants. SL has been involved with the MPDA for 24 years and her Masters’ degree focused on PwP.

### Participants and recruitment

PwP and their caregivers who were members of MPDA were recruited via purposive sampling to obtain a target sample size of 12–15 PwP and/or caregivers. An advertisement about the study was displayed in the MPDA and interested participants were asked to get in touch with the researchers. Potential participants were also approached in person by MPDA staff members. To be included, participants had to be 18 years of age and above. Participant information sheets were provided to all participants, and signed consent forms were obtained prior to participation.

### Data collection

The FGD was conducted at the MPDA on a Saturday morning when members had no planned activities. The FGD was conducted by author WLC with assistance from author SAJ, both qualified clinical pharmacist-academics. The session was audio-recorded and field notes were taken to capture key points. Participants were requested to provide some demographic details, and lunch was provided at the end. The interview guide (Appendix 1) was developed based on the study objectives and review of the literature. The FGD was semi-structured; during the FGD the guide questions were asked along with other pertinent and emerging follow-up questions. Topics included challenges faced with the healthcare system and medications, PC needs, and views on a pharmacist-run clinic. Face and content validation of the guide were performed by experts in qualitative research and academics with expertise in PC.

### Data analysis

The recorded FGD was transcribed verbatim, and anonymised prior to analysis. Transcripts, audio recordings and field notes were imported into NVivo 11 software for analysis. Thematic analysis was performed by three researchers (WLC, ZJW and SAJ), guided by Braun and Clarke’s six phase approach to coding [14]. Quotations from participants were edited on a limited basis to remove content that did not convey meaning (repeated words, editing errors etc.) and to correct for grammar.

## Results

**Fig. 1:**
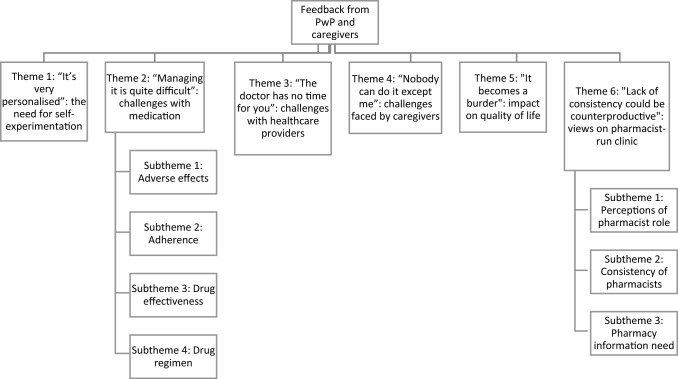
Themes and subthemes

Thirteen participants took part in the FGD, comprising nine PwP and four caregivers. There was a slight male preponderance (61.5%), and the average age of participants was 68.2 years (± SD 9.1) with a range between 51 and 81. The average number of years that PwP had been diagnosed with PD was six (± SD 3.2). All caregivers were spouses of the PwP. The FGD lasted 1 h and 40 min. Six themes from the analysis were developed (Fig. 1).

### Theme 1: “It’s very personalised”: the need for self-experimentation

Several PwP mentioned making self-adjustments to their antiparkinsonian medication in order to achieve better symptom control and predictability of their ‘on/off’ times. They considered it their responsibility to experiment with their medication regimen—taking it upon themselves to discover what worked best for them, irrespective of whether their doctors were aware of the experimentation. A few obtained their doctor’s approval before making adjustments. PwP and caregivers believed their medication regimens had to be personalized, noting *“it’s not one rule for everybody. So experimenting is something you have to do whether it works or not”(Caregiver #2, male).*

Many experimented with the timing of their doses, adjusting it to how they felt symptomatically as well as how their surrounding environment affected them on that particular day. PwP also adjusted the timing of their doses to ensure they had symptom control when they most required it e.g. when they were busiest with their daily activities. Adjustments were also made after observing differences in how fast and how well their medication worked when taken at different times relative to their mealtimes.

There was great curiosity about why each person might respond differently and why they kept having to make adjustments as the disease progressed. They also admitted that the results of their experimentation with drug dosages were often inconsistent and did not provide the same effect every time, necessitating more self-adjustments over time:

*“I think like all of us here, we all experiment right? […] and the conclusion really is you don’t know […] you can do the same thing for one week, two weeks, three weeks, and you’ll have different results.”* (PwP #6, female)

### Theme 2: “Managing it is quite difficult”: challenges with medication

#### Subtheme 1: Adverse effects

Adverse drug reactions (ADRs) such as dry mouth, dizziness, speech difficulties, constipation, and drowsiness, were among the most common challenges mentioned by PwP. One described how she suffered from dry mouth after taking Benzhexol (trihexyphenidyl), which her doctor failed to inform her about. Speech difficulties and stammering were also attributed to ADR, with one noting: *“…I don’t speak like this. It’s very difficult for my words to come out from my mouth because of medication” (PwP #5, male)* Although their doctors were working with them to identify the medications causing this effect, information on the next course of action was not provided to them. There was frustration among the PwP experiencing this problem, and this was one of the occasions where they felt that they had to adjust their medication regimen in order to avoid developing speech difficulties.

PwP also complained of frequently feeling sleepy and dozing off during their daily activities: *“I think I spend about 70% of the time being sleepy.”( PwP #1, female).* Potential antiparkinsonian ADRs also affected PwP’s willingness to use them. For example, a few PwP cited dyskinesia due to Levodopa as a major reason why they avoided taking it. They were also concerned about the long-term effects of taking their antiparkinsonians.

#### Subtheme 2: Adherence

Adherence was highlighted as a common challenge by PwP. One with advanced PD who was on multiple medications up to three times a day mentioned that he tended to either take his medication later than scheduled or forget them completely. The PwP’s emotional state, such as being excited and distracted, could also cause them to forget their medication:

*“Because if it is a good day, she feels quite happy that day, [if there’s] some event coming up […], my sister comes from London…[she gets] very excited […] you can forget about the medicine, you know”* (Caregiver #2, male).

For some, forgetting to take their medication was of no great consequence, saying *“[If] you miss a dose [it’s] no problem. So it is not a [medicine you] must take…”* (Caregiver #2, male).

#### Subtheme 3: Drug effectiveness

Some PwP and caregivers voiced their doubts about the effectiveness of their antiparkinsonians. Much of the frustration arose from the often-unpredictable onset and duration of action of their antiparkinsonians. This unpredictability meant that PwP were unable to correctly time their doses to avoid the ‘off’ state, leading to movement difficulties and “freezing”. The term “drug failure” was frequently used by PwP when the medication was less effective and took longer than expected to relieve their symptoms:

*“I also face a lot of problem because of drug failure. For example, today I took [it] at 10 o’clock [am] and until now I’m still not feeling the drug effect.”* *(PwP #1, female)*

#### Subtheme 4: Drug regimen

Pharmaceutical burden complicated the management of their PD, as most PwP reported having comorbidities, which they also had medication for. This caused confusion as to when they should take both their PD and non-PD medication:

*“…for Parkinson’s alone, it’s already five different types plus her injection […] she also has […] very serious constipation…also takes […] laxatives for constipation. She also has severe osteoporosis […] so she also has to take medication for that […] she also takes medication for dementia. Oh my goodness! […] so managing it is quite difficult…scheduling is one concern”* (Caregiver #2, male)

PwP were also confused as to whether they should follow their doctors’ instructions strictly, or make their own adjustments. The need to take their medications as frequently as every 2 h was also a source of frustration.

### Theme 3: “The doctor has no time for you”: challenges with healthcare providers

Poor patient-provider communication was a source of frustration, where they often felt unheard by their doctors. One related how one of the doctors insisted that they adhere to the previously prescribed medication regimen, even though the PwP had complained about the lack of efficacy.

Some PwP also encountered doctors with negative perceptions about PD. A PwP related her disappointment at being told by her doctor that PD was an incurable disease and that all she could do was count the days to her death. Another was similarly disappointed when his doctor did not address his medication issues, but instead remarked that he should be grateful that he had managed to live for as long as he had. As a result, some PwP confessed that they had given up on telling their doctors about the problems they faced because they assumed that their doctors were either too busy or uninterested:

*“If you are going to discuss with your doctor, this is my experience, don’t go and consult him […] he has no time for it. What you do is […] you experiment…and say “Hey doc, I am doing this now for my wife.” […] Is it okay?” […] You don’t go and ask him “what should I do?” He has got no time for you.” *(Caregiver #2, male)

The challenges in communication also led to a loss of confidence in their doctors. As a result, PwP relied on themselves to get the information they needed. When one caregiver asked the doctor if the medicine can be taken long-term, the doctor responded *“I think so.”* This shocked the caregiver who shared *“My goodness! […] [I] almost dropped from my chair […] Then you have to go back and do research on your own…*” (Caregiver #2, male).

With regard to pharmacy services, participants lamented that in most cases, they did not receive any counseling or useful information about their medication, specifically on its role and effect in managing their disease. Participants also reported that their pharmacists often did not understand their questions and were unable to provide answers:

*“You talk to doctors, either they say “Don’t understand, can’t tell you or too busy to tell you. You talk to [a] pharmacist”. I think most of the pharmacist also would not [know] because this is a complicated case, it’s not just taking one type of medicine. You [are] taking about seven, eight different types of medicine, I think you [will] make the pharmacist go cuckoo also…”* (Caregiver #2, male)

Several participants expressed their concern about not knowing who and where to seek answers from. As a result, they turned to health magazines and online resources.

### Theme 4: “Nobody can do it except me”: challenges faced by caregivers

Caregivers of those with advanced PD expressed their burden of being unable to leave their charges, and having to be with them throughout the day due to their complicated medication regimen. Experimenting was challenging as it was difficult for them to judge if the treatment was working effectively, as some PwP had communication difficulties:

*“When you experiment...you have to guess. Is it working, is it not working? […] So that makes it even more difficult because especially older people sometimes they are disoriented and […] bed ridden, they can’t actually think properly. You ask them how are they feeling, and they can’t tell you whether the medicine is working or not...”* (Caregiver #2, male)

### Theme 5: “It becomes a burden”: impact on quality of life

Participants discussed their quality of life. This was an unexpected focus of the FGD yet prompted the interviewees to ask a follow up ad-hoc question whereby participants were asked how PD and their drugs affected their quality of life from a scale of ‘1-10’. Most PwP selected the lower end of the scale. Some of the reasons were because they felt they were a burden to their family members who were now responsible for them. PwP also experienced the loss of work productivity which led to financial constraints, forcing other family members to assume the role of breadwinner: *“…when you see your family you are putting [a] burden on them because they have to take care of you […] so where is the quality of life for me?” (PwP #4, male)*

Being unable to drive due to their illness also negatively affected their quality of life, with some PwP mentioning that they missed driving as they now had to rely on their caregivers to get around. They also attributed their low quality of life to the ADR of their medication and the nature of the disease itself. One PwP lamented that as the disease progressed, there were more ‘off’ times instead of ‘on’ times, affecting her daily activities:

*“And I think the tragedy is as time goes on...you will get more ‘off’ time than ‘on’ time…it’s quite terrible when you’re off because you’re not too sure when you will be okay again. And you can’t plan your schedule and usual activities…”* (PwP #8, female)

The caregivers’ responses were more evenly spread across the scale, with some commenting that it was a privilege to be able to care for their loved ones. Others lamented the loss of the active lives they once had. Both PwP and caregivers felt their quality of life declined when they were not socializing with others, with one stating: *“…if we remain in the house and don’t interact […] I think our mood will go down. You see our quality will also go down.” (#Caregiver #3, male).*

### Theme 6: “Lack of consistency could be counterproductive”: views on pharmacist-run clinic

#### Subtheme 1: Perceptions of pharmacist role

There was a lot of skepticism about the role of pharmacists, with PwP expressing concerns that pharmacists would “overstep” their roles as “dispensers” and a source of drug information. When asked what services they would like provided by the pharmacist in the pharmacist-run clinic, responses were limited to dispensing duties and providing information:

*“Pharmacists only […] delivers the medicine […] the pharmacist cannot explain to you […] the medical side because in this country there is a division. So…you got to be careful. When the pharmacist is starting to become a doctor then you have a problem. The doctor will always give you the treatment and the medication to take it. Pharmacist dispenses and advises the patient.”* (PwP #4, male)

Furthermore, there were concerns that the information provided by the pharmacist would differ from that provided by the doctor.

#### Subtheme 2: Consistency of pharmacists

Participants also wanted to have regular pharmacists instead of a short-term rotating roster of pharmacists so they could spend more time addressing their current issues and their medication:

*“They come and see different people over different times, then they have to tell their story all over again. It’s going to be a problem...on many occasions [the session is] about the patient’s history and not about treating the current condition.”* (Caregiver #4, male)

#### Subtheme 3: Pharmacy information need

There were a lot of misconceptions and doubts about how antiparkinsonians worked. Both PwP and caregivers also had questions about the expected ADRs and how to manage them, and the factors affecting the duration and effectiveness of their antiparkinsonians. Confusion was also expressed with regard to the dosing of the antiparkinsonians, *“Then what is the time the medicine is supposed to work? [If] it doesn’t work then what are [we] supposed to do?” (PwP #1, female).* Both PwP and caregivers frequently expressed the need for reliable and trustworthy information regarding their PD and the drugs provided to them. Participants also expressed the desire for follow-up information regarding drugs, instead of the same advice being given every time they saw the pharmacist.

*“I think the most important thing is providing […] advice and information […] in regard [to] contraindication, side effects…quite good if you can occasionally update people about developments in the pharmaceutical area.”* (Caregiver #2, male)

## Discussion

Findings from this study revealed that PwP and their caregivers have many concerns about their medication and disease, expressing uncertainties about how to manage both. This is compounded by challenges faced with the healthcare system such as communication barriers with HCPs, that lead to self-experimentation without their doctor’s knowledge. Other challenges include ADRs and high pill burden, both of which had a negative impact on medication adherence. These factors also contributed to the low quality of life reported. There was scepticism about the role the pharmacist would play in the proposed pharmacist-run clinic as well as doubts about the expertise of pharmacists.

It has been postulated that people with incurable chronic diseases such as PD, resort to unsupervised self-experimentation, which they feel is a ‘legitimate form of treatment for incurable diseases’ [15]. Participants in this FGD, however, cited drug ‘failure’ or ineffectiveness as the main reason for their frequent experimentation. This can be ascribed to their lack of knowledge about the nature of PD, a complex, ‘multisystem, multineurotransmitter dysfunction-related heterogeneous disorder’ [16, 17] resulting in significant variability between individuals.

The majority of participants in this study experienced ADRs, and stated that they were also not informed by their doctors about possible ADRs. This has been noted in other studies [18], and can be ascribed to fear on the part of HCPs that knowledge on ADRs would deter people from taking their medication [19]. However, having good knowledge on medication and its effects has a positive effect on adherence [18, 20, 21]. Patients themselves have expressed a desire for more information from their HCPs [19, 22, 23], especially about the ADRs of a drug, as illustrated in a survey of more than 2000 respondents, where close to 80% wanted information on all possible side effects of a drug [24]. The knowledge of what to expect and how to deal with it increases confidence and was seen as an independent predictor of adherence [20]. In the same study, participants also found ADRs more tolerable if they had been informed about them, thus increasing the probability of them being adherent [25, 26].

One of the facilitators of adherence is good communication between patients and their HCPs, with studies showing it had a profound impact on patients’ willingness to adhere to treatment [2, 4]. Participants in this FGD, however, have described a fractured patient-doctor relationship, attributing this to the lack of time by doctors and what they believed to be a lack of knowledge and/or poor attitude on the part of doctors with regard to PD. Thus, participants complained about not being given enough information, which is similar to that reported in other studies [22].

The limited view of the pharmacist and pharmacist-led services by PwP expressed in this study is also reflected elsewhere in the literature. In Malaysia, participants of a qualitative study viewed pharmacists mainly as suppliers of medication and had trouble envisaging their expanded roles as part of a healthcare team [27]. There is indeed an erroneous perception that a pharmacist’s main role is dispensing, with many unaware of the enhanced roles of pharmacists such as providing PC services and managing long-term diseases [28–36] It has been postulated that this ‘reductionist perception’[36] of pharmacists stems from the lack of interaction between patients and pharmacists at a higher level [35]. Where there is a lack of understanding of these expanded pharmacist roles, pharmacists can find it challenging implementing services and the ability of the profession for role expansion can be hindered [33].

### Recommendations and future research

Pharmacists must be seen working side-by-side with other HCPs as part of a multidisciplinary team and in their rightful role as experts in medications and PC in specialist clinics. This will help allay peoples’ fears about pharmacists managing their medication, as they will see that pharmacists will not be working in isolation, but as part of a multidisciplinary team. This will also help them see that pharmacists can act as a bridge to facilitate transfer and sharing of info, and to act as a consultant to assist doctors in individualising therapy [16]. A study in the United States found that the addition of a pharmacist, who provided PC services, to the outpatient multidisciplinary team resulted in patients having an improved understanding of their medications. The presence of the pharmacist was rated highly by both patients and HCPs, with the latter saying it gave them more time to focus on other clinic responsibilities [37]. Similarly a study in Malta found that pharmacists involvement in the outpatient setting resulted in a significant improvement in adherence rates and quality of life, and more than 80% of patients felt that the pharmacist’s role was integral in managing the disease [38].

The successful design and implementation of a pharmacist-led service for PwP and their caregivers should be guided by evidence gathered from the local population. This study sought to generate evidence from the perspective of PwP and their caregivers. Future studies should explore the perception and attitudes of Malaysian pharmacists towards the needs and challenges faced by this population, as well as their views on how a pharmacist-led service would benefit PwP and their caregivers. Similarly, studies aimed at understanding the perspective of the PwP’s neurologists and physicians will also provide valuable information. Evidence from multiple stakeholders will allow for the design and implementation of a pharmacist-led service that is acceptable and culturally-appropriate for the local Parkinson’s population.

In Malaysian public hospitals, pharmacists undertake activities such as patient education and counselling, solving DRPs, and monitoring disease progression in pharmacist-managed clinics known as Medication Therapy Adherence Clinics (MTAC) [39, 40].These MTACs have resulted in significantly better clinical outcomes compared to usual care [41]. As such, evidence exists that suggests a similar service might benefit PwP and their caregivers. We therefore recommend that a trial pharmacist-led service be conducted with the cooperation of local PwP support groups in order to evaluate the potential impact and benefit of the service as well as any adaptations that need to be made for this population.

Campaigns to promote the role of pharmacists should also be undertaken, similar to that launched by the Royal Pharmaceutical Society in the United Kingdom which resulted in the public having a more positive perception of pharmacists and utilizing expanded services offered by them [42]. Research should also be undertaken to inform public health messaging about the role of pharmacists.

### Strengths and limitations

This is the first study to be conducted involving both PwP and caregivers, which looked at the healthcare challenges faced. The use of qualitative methods also allowed for more in-depth exploration around issues that mattered to participants, which allowed for unexpected emergent themes such as quality of life. In addition, a multidisciplinary team was involved in the development of the study guide as well as the conduct of the study. The FGD involved both caregivers and PwP and we see this as a strength as both groups play central roles in medication and disease management, and through the interaction and discussion between caregivers and PwP, the researchers were able to obtain a picture of participants’ knowledge, as well as attitudes and beliefs toward the medication and disease.

Participant recruitment was limited to members from the Malaysian Parkinson’s Disease Association, which is located in an urban area. Thus, the views expressed by these participants might not be representative of those living in rural areas or from lower socioeconomic backgrounds, who might face other challenges. Only one FGD was conducted therefore saturation of themes was not guaranteed. The purpose of the FGD was to inform the design of the pharmacist-run clinic therefore we do not claim the reported thematic outcomes to be established research findings. The findings, nonetheless, raise valuable information about how PwP and their caregivers are managing their medications and disease, and will be useful to healthcare providers. The findings can also and should be used as a precursor to either conducting more FGDs or developing a survey to be distributed to PwP and caregivers.

## Conclusions

PwP and their caregivers face numerous healthcare challenges, the bulk stemming from gaps in knowledge and communication. While pharmacists could assist PwP in PD management, there is currently a poor understanding of their expanded roles. This can be overcome by integrating pharmacists within specialist clinics so PwP can see them undertake these expanded roles within a multidisciplinary care team.

## Appendix 1

### Interview guide

1. What are the challenges with accessing pharmacists/pharmacy-services in the community or hospital
2. Are there any communication problems with doctors/nurses? If so, what are they?
3. Are there any communication problems with pharmacists? If so, what are they?
4. What issues do you currently face with your medication, both PD and non-PD?
5. Are they are other common needs related to medication for diseases other than your PD such as hypertension, asthma, diabetes etc.?
6. Do you feel you fully understand the role of different medicines in managing PD?
  - Do you think it is necessary for you to know these things?
7. What are your counselling needs, specific to medications
8. What are the challenges caregivers have with managing the medicines of PD patients
9. Do you think there is a need for a pharmacist-run clinic within MPDA?
  - What type of services would you like to see offered at this clinic? E.g. BP check, glucose, cholesterol check etc.
  - Would you be willing to pay for such a service especially as it pertains to glucose and cholesterol checks to cover the cost of the strips?
10. Would you want doctors and/or nurses to be involved with the clinic?

## Appendix 2

COREQ (COnsolidated criteria for REporting Qualitative research) 32 item Checklist.

Tong A, Sainsbury P, Craig J. Consolidated criteria for reporting qualitative research (COREQ): a 32-item checklist for interviews and focus groups. International Journal for Quality in Health Care. 2007. Volume 19, Number 6: pp. 349–357.

**Table:**

Domain	Comment	Section reported in or not applicable (N/A)
Domain 1: Research team and reflexivity		
*Personal characteristics*		
1. Interviewer/facilitator	WLC conducted the focus group discussion (FGD) with assistance from SAJ. Both were pharmacy-qualified academics	Method
2. Credentials	The research team was made up of two pharmacy-qualified academics, one who was a board-certified pharmacotherapy specialist with a special interest in the management of patients with neurological disorders (WLC), and the other who had a doctorate in clinical pharmacy (SAJ). The others were two undergraduate pharmacy students (ZJW and YXW) and a pharmacist (EYC)	N/A
3. Occupation	WLC and SAJ were academics, ZJW and YXW were undergraduate pharmacy students, and EYC was a pharmacist	N/A
4. Gender	Four female-identifying researchers, and one male-identifying researcher. The main interviewer was male-identifying, while the assistant was female-identifying	N/A
5. Experience and training	Three members of the team were experienced in qualitative research and studies focusing on pharmaceutical care (EYC, WLC and SAJ)	N/A
*Relationship with participants*		
6. Relationship established	SAJ had previously had worked with the MPDA to organise service-learning placements for undergraduate pharmacy students. None of the researchers were involved in the care of the participants or interacted with the participants prior to the study We felt the main interviewer (WLC) should be seen as an unbiased enquirer	N/A
7. Participant knowledge of the interviewer	Interviewer credentials were available on the participant information sheet handed out to participants. Prior to the start of the FGDs, the interviewer informed participants about the objectives of the study and answered any questions participants may have had about the study as well as those involved in it	N/A
8. Interviewer characteristics	The interviewer (WLC) previously practiced as a clinical pharmacist at the Institute of Neurosciences in a government hospital in Malaysia, and had interactions with some of the doctors who treated the participants	N/A
Domain 2: Study design		
*Theoretical framework*		
9. Methodological orientation and theory	A descriptive qualitative research design was used	Methods
Participant selection		
10. Sampling	Purposive sampling was used	Methods
11. Method of approach	An advertisement about the study was put up in MPDA and interested participants were asked to get in touch with the researchers. Potential participants were also approached in person	Methods
12. Sample size	One FGD consisting of 13 participants	Results
13. Non-participation	No participant dropped out once agreeing to take part	N/A
*Setting*		
14. Setting of data collection	The FGD was conducted at the MPDA	Method
15. Presence of non-participants	SL, the president of the MPDA who was involved in the development of the interview guide and recruitment of study participants was present	Method
16. Description of sample	Fully presented in results section	Results
*Data collection*		
17. Interview guide	An interview guide was developed based on the study objectives ad review of the literature, and validated by experts in qualitative research and pharmaceutical care	Method
18. Repeat interviews	No	N/A
19. Audio/visual recording	The session was audio recorded	Method
20. Field notes	Field notes were captured during the session to capture key points and make notes on any perceptions with regard to participants’ responses	Method
21. Duration	The FGD lasted 100 min	Results
22. Data saturation	Only one FGD was conducted	N/A
23. Transcripts returned	No	
Domain 3: analysis and findings		
*Data analysis*		
24. Number of data coders	Coding and thematic analysis were performed on the transcripts by three researchers (WLC, ZJW and SAJ)	Method
25. Description of the coding tree	A description of the coding tree is not provided. Only themes and subthemes generated are presented	
26. Derivation of themes	Themes were derived from the data	Method
27. Software	NVivo	
28. Participant checking	None	
*Reporting*		
29. Quotations presented	Yes quotations are presented. Anonymized participant details are used for each quotation	Results
30. Data and findings consistent	Yes there is consistency between data and findings	Results
31. Clarity of major themes	Major themes are clearly illustrated	Results
32. Clarity of minor themes	Minor themes are also highlighted	Results
